# Reliability of finger strength assessment methods in climbing: a systematic review

**DOI:** 10.3389/fspor.2025.1650198

**Published:** 2025-10-01

**Authors:** Jorge Pérez-Cordero, Daniel Jerez-Mayorga, Ángela Rodríguez-Perea, Diego Soto García

**Affiliations:** ^1^ Faculty of Physical Activity and Sports Sciences, University of León, León, Spain; ^2^ Department of Physical Education and Sports, Faculty of Sport Sciences, Sport and Health University Research Institute (iMUDS), University of Granada, Granada, Spain; ^3^ Faculty of Rehabilitation Sciences, Exercise and Rehabilitation Sciences Institute, Universidad Andres Bello, Santiago, Chile; ^4^ Department of Physical and Sport Education, Faculty of Physical Activity and Sports Sciences, Universidad de León, León, Spain; ^5^ Strength & Conditioning Laboratory, CTS-642 Research Group, Department of Physical Education and Sport, Faculty of Sports Sciences, University of Granada, Granada, Spain; ^6^ Department of Physical Education and Sports, University of León, León, Spain

**Keywords:** sport climbing, finger flexor strength, grip strength, maximal isometric finger strength (MIFS), test–retest reliability, reproducibility, performance assessment, systematic review

## Abstract

**Background:**

This systematic review examined the reliability of finger flexor strength assessments in climbers, addressing the absence of a prior synthesis on this topic. The work is timely given sport climbing's inclusion in the Olympic Games and the growing focus on sport-specific performance diagnostics. Fifteen studies, comprising 747 participants (sample sizes 13–244) with varying skill levels, were included.

**Methods:**

Conducted in accordance with PRISMA guidelines and based on a protocol registered in INPLASY, the search encompassed Web of Science, PubMed, Scopus, and SportDiscus, using MeSH terms and relevant keywords. Eligible studies involved climbers, employed a test–retest design, reported strength variables, and provided reliability parameters (ICC). Methodological quality was evaluated with the Critical Appraisal Tool (CAT) and the Quality Appraisal for Reliability Studies (QAREL).

**Results:**

Fourteen studies reported high reliability (ICC > 0.75) in at least one assessment, while 12 studies showed very high ICC values for maximum isometric finger strength (MIFS) tests (median range: 0.85–0.99), indicating good to excellent reliability. Most studies (*n* = 12) used varied grip types and edge depths (6–60 mm). Bilateral measurements were included in eight studies, though five used non-simultaneous protocols, potentially limiting ecological validity.

**Discussion:**

Adoption of advanced measurement technologies and harmonized protocols is recommended to enhance comparability, practical relevance, and training effectiveness. These measures may also contribute to greater standardization in research designs and facilitate translation of findings into applied settings.

**Conclusion:**

MIFS assessments with fixed-depth edges of approximately 20–23 mm consistently demonstrate high reliability and should be prioritized for standardized monitoring in both applied and research contexts.

**Systematic Review Registration:**

https://inplasy.com/inplasy-2024-10-0070, identifier INPLASY2024100070.

## 1 Introduction

In recent years, the sport of climbing has experienced exponential growth in the number of participants, both at competitive and recreational levels. This surge in popularity, especially following its inclusion in the Tokyo 2020 Olympic Games, has significantly increased its prominence and prestige, leading to a larger pool of participants across various competitive levels (1). This growth has brought with it challenges, such as the prevalence of injuries, particularly among elite female climbers, and the need for injury prevention strategies (2). Consequently, interventions focused on performance enhancement and injury prevention are essential. Maximal isometric finger flexor strength (MIFS) is a key determinant of climbing performance, showing a strong correlation with grip capacity, particularly when expressed relative to body mass (3–6). The half-crimp (HC) position has been identified as a major performance predictor, explaining much of the difference between advanced and elite climbers (7). In addition, the rate of force development (RFD) and maximal upper-body strength clearly distinguish elite climbers from those at lower performance levels (8). Therefore, objectively assessing MIFS provides a quantifiable way to evaluate a climber's physical potential and pinpoint specific areas for improvement. Given its relevance to both performance optimization and injury risk reduction, MIFS assessment represents a cornerstone for evidence-based training and health preservation in climbing. Strength metrics do far more than quantify an athlete's current capabilities; they provide a solid analytical basis for developing training strategies that address the specific physical and technical demands of climbing (9, 10). In this context, bridging the gap between physiological assessment and practical application becomes essential, ensuring that strength evaluations translate into actionable strategies for safer and more effective performance (a point particularly relevant for enhancing ecological validity and practical applicability in real-world climbing scenarios). In the past decade, advances in portable, sensor-based technologies have not only improved the precision of performance assessment but have also made it possible to conduct meaningful evaluations in real climbing environments, beyond the confines of laboratory settings (11, 12). The capacity to measure grip strength across different grip types and body positions is essential for promoting progressive physical development and for protecting athletes from excessive loading or preventable injuries (13). Both commercial and specialized force sensors have consistently demonstrated high reliability for climbing-specific strength testing (14). Despite the growing use of these tools, no previous synthesis has systematically compared the reliability of the full range of finger flexor strength assessment methods used in climbing. Addressing this methodological gap is critical, not only for refining performance prediction models and evaluating the effectiveness of training interventions, but also for embedding injury prevention strategies as a central element of climbing preparation (10, 15). Despite the variety of available tests, the lack of standardization complicates comparisons and recommendations, emphasizing the need for more uniform test batteries for evaluating climbing strength (16). This review therefore aims to examine, in a structured and critical way, the methods currently available for assessing finger flexor strength in climbers, highlighting both their methodological robustness and their practical implications for performance and safety.

## 2 Materials and methods

We conducted a systematic review to determine the reliability of finger strength assessment tests in climbers of all levels, from recreational participants to elite competitors, spanning IRCRA (17) levels 14 to 32. All included studies employed a test-retest design, with evaluations measured using the Intraclass Correlation Coefficient (ICC). Both quantitative and qualitative summaries were included: (a) a quantitative analysis of key study variables, and (b) a qualitative review of factors influencing reliability and their relationship with performance. Before starting the review, a protocol was registered on the International Platform of Registered Systematic Review and Meta-analysis Protocols (INPLASY) under the registration number INPLASY2024100070. The systematic review followed the PRISMA flow diagram guidelines and adhered to the best practices outlined in the guidelines for systematic reviews in sports sciences (18).

### 2.1 Literature search strategy

Relevant studies were identified through searches in the primary databases: Web of Science, PubMed, Scopus, and SportDiscus. The literature search combined Medical Subject Headings (MeSH) terms with keywords such as “Reliability”, “Reproducibility”, “Rock Climbing”, “Sport Climbing”, “Boulder”, “Lead Climbing”, “Climbers”, “Dynamometer”, “Finger Strength”, and “Handgrip Strength”. The search terms were combined using the Boolean operators AND and OR. Two authors (J.P. and D.J.) reviewed the titles and abstracts of all articles identified in the various databases. Following this preliminary selection, the studies were analyzed according to the inclusion and exclusion criteria. Five inclusion criteria were established and evaluated on a yes/no basis. No discrepancies were found between the authors, and no differential bias was detected in the studies selected for inclusion in this review. The literature was screened from inception to January 31, 2024, with eligibility restricted to studies published in English or Spanish, with records managed in Rayyan, where duplicates were removed using automated detection of identical titles, authors, publication year, and DOI, followed by manual verification to ensure accuracy. To ensure transparency and reproducibility, the complete search strings for each database are provided in the Supplementary Material S1 (Excel file).

### 2.2 Eligibility criteria

Original research was deemed eligible for inclusion in the systematic review if it met the following criteria: (a) participants were climbers; (b) studies employed a test–retest design; (c) studies reported strength-related variables; (d) studies provided a reliability parameter (ICC); and (e) studies utilized a device to assess muscle strength. Studies with significantly different methodologies that could affect result comparability were excluded even if they met the inclusion criteria. For greater methodological clarity, these criteria were framed using the PICOS approach: the population was climbers, the interventions were protocols to assess finger flexor strength, the comparison was test–retest reliability, the outcomes were reliability indices (primarily ICC), and the eligible design was original test–retest studies.

### 2.3 Data extraction and study characteristics

The following data were extracted from each selected article for the review: number of participants, gender, type of participants (climbers), unilateral or bilateral evaluation (hands), simultaneous or non-simultaneous testing, rest intervals between tests, devices used for measurements (including dynamometers, if applicable), protocol, reliability (ICC), edge depth, hand grip, and whether environmental conditions during the tests were recorded. Because most studies did not report the ICC model or form used, we extracted the main ICC values exactly as provided by each study. When multiple ICCs were reported for the same protocol, we used either the value explicitly identified by the authors as the primary outcome or, if none was specified, the median ICC across test conditions. This approach ensures transparency while acknowledging the lack of standardized ICC reporting across studies. Data extraction was carried out by three authors (JP, DS, and DJ) using forms designed in advance following PRISMA guidelines. Each reviewer worked independently, and afterwards, all four authors (JP, DS, DJ, and AR) reviewed the dataset together and resolved any differences by consensus. Studies with methodological protocols that differed substantially in test execution (e.g., non-isometric assessments, evaluation of arm strength, or other protocols not directly targeting finger flexor strength) were excluded, as these would prevent meaningful comparison of reliability outcomes.

### 2.4 Methodological quality assessment

The methodological qualities of the selected studies were evaluated using the Critical Appraisal Tool (CAT) (19) and the Quality Appraisal of Reliability Studies (QAREL) (20). The CAT scale consists of 13 items assessing both reliability and validity; however, because this review focused exclusively on reliability outcomes, only the 9 items directly related to reliability were analyzed, while validity-related items were excluded since instrument validity data were inconsistently reported across studies. This approach minimized uncontrolled variability and ensured that final quality scores reflected methodological rigor in reliability assessment alone. The QAREL scale contains 11 items: items 1–2 address sample bias and participant representativeness; items 3–7 evaluate evaluator blinding; item 8 concerns the order of subject evaluations; item 9 assesses the interval between repeated measurements; item 10 evaluates whether the test was appropriately applied and interpreted; and item 11 addresses statistical analysis (20). Final quality scores were expressed as percentages, with 90% indicating the highest methodological quality and scores above 45% considered indicative of high-quality studies, following criteria from previous methodological reviews in sports science. We acknowledge that while CAT and QAREL are widely used for methodological appraisal, they have inherent limitations when applied specifically to reliability studies; therefore, these quality ratings should be interpreted cautiously, and the conclusions of this review consider such constraints.

### 2.5 Data collection and synthesis

To minimize bias and ensure objectivity, the information extracted was organized systematically into an Excel database and analyzed with the help of Rayyan. Data were categorized into three groups: Participant Characteristics (Table 3), Methodological Aspects (Table 4), and Variables of Interest and Key Results (Table 5). This categorization facilitated qualitative synthesis of the findings. Given the pronounced methodological heterogeneity across studies (differences in grip type, edge depth, devices, and testing protocols), a formal meta-analysis was not conducted. Instead, results were synthesized narratively and, where possible, grouped by common methodological characteristics (e.g., grip type, edge size, or measurement device) to allow more meaningful comparison.

### 2.6 Ethics

This systematic review did not require ethical approval as it involved analysis of previously published data. All included studies were conducted in accordance with the ethical standards of the respective institutional and/or national research committees and with the 1,964 Helsinki Declaration and its later amendments.

## 3 Results

**Figure 1:**
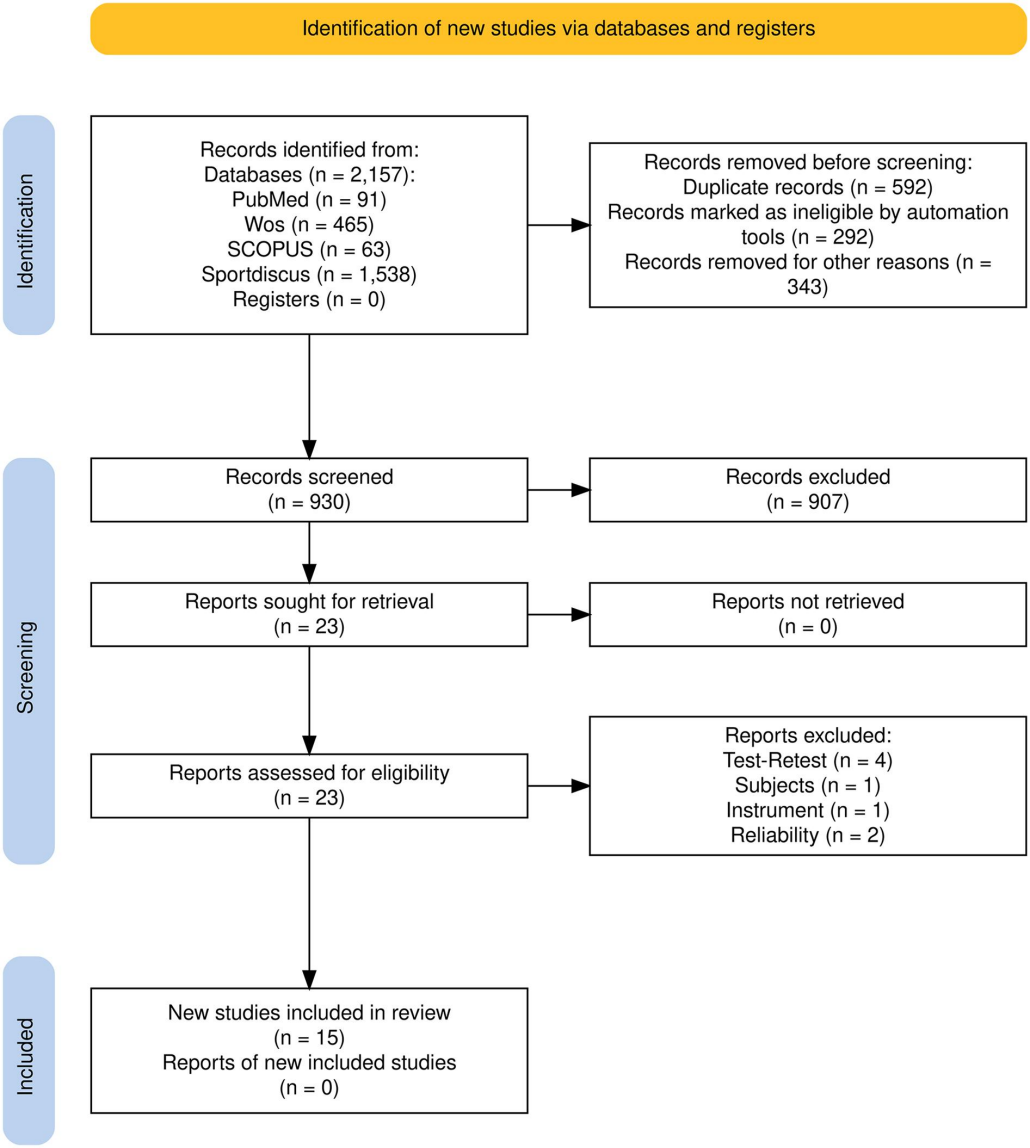
PRISMA 2020 flow diagram of the study selection process.

Study Selection (Figure 1).

An initial electronic search identified 2,157 potentially relevant references from the databases (PubMed, *n* = 91; WoS, *n* = 465; SCOPUS, *n* = 63; Sportdiscus, *n* = 1,538). After removing 1,227 duplicates, 907 articles were excluded after reviewing their titles and abstracts. This left 23 studies for full-text review, of which eight were excluded for the following reasons: four did not include a test-retest design, one targeted a population outside the scope of the review, one did not use measurement instruments, and two did not report reliability data. Ultimately, 15 studies met the inclusion criteria. However, substantial variability was observed in the grip types (21), edge depths, and protocols used, resulting in widely varying Intraclass Correlation Coefficient (ICC) values, even within individual studies. Across all studies, ICCs ranged from 0.294 to 1, with a median value of 0.86. This marked heterogeneity in the reported data and study designs precluded the possibility of conducting a meta-analysis.

“The study selection process is illustrated in the PRISMA 2020 flow diagram (Figure 1)”.

### 3.1 Risk of bias in studies

CAT scores ranged 44.4%–55.5%, while QAREL scores ranged 36.46%–45.45% (Tables 1, 2). Scores below 50% on either scale were interpreted as moderate methodological quality, reflecting incomplete reporting or potential bias in areas such as evaluator blinding, standardization, or environmental control. Higher ICC values tended to be reported in studies with higher CAT scores, although this pattern was not consistent across all comparisons.

**Table 1:** Evaluation of study quality using clinical evaluation tool (CAT).

Study	1	2	3	4	5	6	7	8	9	Total (%)
Morenas et al. (22)	Y	N	NA	NA	N	Y	Y	N	Y	44.4%
Watts and Jensen (23)	Y	N	NA	NA	Y	Y	Y	N	Y	55.5%
Mcclean et al. (24)	Y	N	NA	NA	Y	Y	Y	N	Y	55.5%
Bergua et al. (25)	Y	N	NA	NA	Y	Y	Y	N	Y	55.5%
Orth et al. (26)	Y	N	NA	NA	Y	Y	Y	N	Y	55.5%
Levernier and Laffaye (27)	Y	N	NA	NA	Y	Y	Y	N	Y	55.5%
Michailov et al. (28)	Y	N	NA	NA	Y	Y	Y	N	Y	55.5%
Torr et al. (6)	Y	N	NA	NA	Y	Y	Y	N	Y	55.5%
Baláš et al. (29)	Y	N	NA	NA	Y	Y	Y	N	Y	55.5%
Baláš et al. (30)	Y	N	NA	NA	Y	Y	Y	N	Y	55.5%
Baláš et al. (31)	Y	N	NA	NA	Y	Y	Y	N	Y	55.5%
Labott et al. (14)	Y	N	NA	NA	Y	Y	Y	N	Y	55.5%
Macdougall et al. (32)	Y	N	NA	NA	Y	Y	Y	N	Y	55.5%
López Rivera et al. (33)	Y	N	NA	NA	NA	Y	Y	N	Y	44.4%
Söderqvist et al. (7)	Y	N	NA	NA	N	Y	Y	Y	Y	55.5%

Y, Yes; N, No; NA, not applicable.

(1). If human subjects were used, did the authors provide a detailed description of the sample of subjects used to perform the test? (2). Did the authors clarify the qualification or competence of the rater(s) who performed the test? (3). If interrater reliability was tested, were raters blinded to the findings of other raters? (4). If intrarater reliability was tested, were raters blinded to their own prior findings of the test under evaluation? (5). Was the order of examination varied? (6). Was the stability (or theoretical stability) of the variable considered when determining the suitability of the time interval between repeated measures? (7). Was the execution of the test described in sufficient detail to permit replication of the test? (8). Were withdrawals from the study explained? (9). Were the statistical methods appropriate for the the study? %: final percentage of reliability (Items “yes”x100)/9.

**Table 2:** Evaluation of study quality using the QAREL scale.

Study	1	2	3	4	5	6	7	8	9	10	11	Total (%)
Morenas et al. (22)	Y	NA	NA	NA	NA	NA	NA	Y	N	Y	Y	36.36%
Watts and Jensen (23)	Y	NA	NA	NA	NA	NA	NA	Y	Y	Y	Y	45.45%
Mcclean et al. (24)	Y	NA	NA	NA	NA	NA	NA	Y	Y	Y	Y	45.45%
Bergua et al. (25)	Y	NA	NA	NA	NA	NA	NA	Y	Y	Y	Y	45.45%
Orth et al. (26)	Y	NA	NA	NA	NA	NA	NA	Y	Y	Y	Y	45.45%
Levernier and Laffaye (27)	Y	NA	NA	NA	NA	NA	NA	Y	Y	Y	Y	45.45%
Michailov et al. (28)	Y	NA	NA	NA	NA	NA	NA	Y	Y	Y	Y	45.45%
Torr et al. (6)	Y	NA	NA	NA	NA	NA	NA	Y	Y	Y	Y	45.45%
Baláš et al. (29)	Y	NA	NA	NA	NA	NA	NA	Y	Y	Y	Y	45.45%
Baláš et al. (30)	Y	NA	NA	NA	NA	NA	NA	Y	Y	Y	Y	45.45%
Baláš et al. (31)	Y	NA	NA	NA	NA	NA	NA	Y	Y	Y	Y	45.45%
Labott et al. (14)	Y	NA	NA	NA	NA	NA	NA	Y	Y	Y	Y	45.45%
Macdougall et al. (32)	Y	NA	NA	NA	NA	NA	NA	Y	Y	Y	Y	45.45%
López Rivera et al. (33)	Y	NA	NA	NA	NA	NA	NA	Y	Y	Y	Y	45.45%
Söderqvist et al. (7)	Y	NA	NA	NA	N	NA	NA	N	Y	Y	Y	36.36%

Y, yes, complies; N, no, does not comply; UC, unclear; NA, not applicable. (1).Was the test evaluated on a sample of subjects who were representative of those to whom the authors intended the results to be applied? (2). Was the test performed by the raters representing those to whom the authors intended the results to be applied? (3). Were raters blinded to the findings of other raters during the study? (4). Were raters blinded to their own prior findings of the test under evaluation? (5). Were raters blinded to the results of the reference standard for the target disorder (or variable) being evaluated? (6). Were raters blinded to clinical information that was not intended for use in the testing procedure or study design? (7). Were raters blinded to additional cues that were not part of the test? (8). Was the order of examination varied? (9). Was the time interval between repeated measurements compatible with the stability (or theoretical stability) of the variable being measured? (10). Was the test applied correctly and interpreted appropriately? (11). Were appropriate statistical measures of agreement used? %: final percentage of reliability (Items ‘‘yes’’x100)/11.

“The quality of each study was assessed using the Clinical Evaluation Tool (CAT), as shown in Table 1”.

“The QAREL scale was applied to evaluate methodological quality across studies. Detailed scores are presented in Table 2”.

### 3.2 Participant characteristics

The total sample size included 747 participants across the 15 (6, 7, 13, 14, 22–25, 27–33) studies, ranging from 13 to 244 climbers with varying performance levels (IRCRA) (17). Additionally, 9 non-climbers were included in a single study (27) as a control group, in which the gender of the participants was not specified.

“Participant characteristics, including age, sex, climbing level, and sample size, are detailed in Table 3”.

**Table 3:** Participant characteristics.

Study	N	Gender	Ability	Subjects
Morenas et al. (22)	93	Men	>17	Climbers
Watts and Jensen (23)	31	Boys/Girls	10–25	Young climbers
Mcclean et al. (24)	13	Men/Women	14–25	Climbers
Bergua et al. (25)	40	Men/Women	>18	Climbers
Orth et al. (26)	32	Men/Women	15–19	Climbers
Levernier and Laffaye (27)	31^a^	(-)	1–32	Climbers/Non Climbers
Michailov et al. (28)	31	Men	12–25	Climbers
Torr et al. (6)	244	Men/Women	15–29	Climbers
Baláš et al. (29)	32	Men/Women	11.23	Climbers
Baláš et al. (30)	55	Men/Women	8–32	Climbers
Baláš et al. (31)	46	Men/Women	3–26	Climbers
Labott et al. (14)	25	Men/Women	>16	Climbers
Macdougall et al. (32)	15	Men/Women	(-)	Climbers
López Rivera et al. (33)	36	Men/Women	13–27	Climbers
Söderqvist et al. (7)	32	Men/Women	>17	Climbers

Ability: IRCRA reporting scale.

^a^ 9 non-climbers.

#### 3.2.1 Protocol (capabilities)

Of the 15 selected studies, seven (6, 7, 14, 22, 30–32) specifically measured the maximal isometric finger flexor strength (MIFS). In five other studies (23, 24, 26–28) MIFS was combined with assessments of other capabilities, such as Endurance (ED), Rate of Force Development (RFD), and Critical Force (CF). This resulted in 12 studies that included MIFS measurements at some point. The remaining three studies (25, 29, 33) focused on ED. Among these, one study (29) employed an endurance protocol to measure muscle oxygenation using Near-Infrared Spectroscopy (NIRS).

#### 3.2.2 Bilateral measurements

Eight of the analyzed studies conducted bilateral measurements. Of these, five (6, 14, 23, 26, 31) used non-simultaneous protocols, while three (22, 25, 33) employed simultaneous protocols. The remaining seven studies (7, 24, 27–30, 32) performed unilateral measurements. Only three studies employed simultaneous bilateral testing, limiting the ecological validity of their findings.

#### 3.2.3 Instruments

All measurements included some type of evaluation instrument, with most studies using dynamometers to monitor these measurements and edges (gripping surfaces of varying sizes), which could be adjustable or fixed at different depths. Three studies (6, 25, 33) did not use dynamometers or electronic instruments to monitor test data. Instead, one study (25) employed a measurement device during the warm-up phase, another (33) determined measurements based on grip depth until muscular failure, and the last (six) used additional weights for climbers, if necessary, to establish the measurement (6). Seven studies (22, 23, 26–29, 31) used specialized force sensors or dynamometers. Four articles (7, 24, 30, 32) used force platforms or electronic scales, one of which (Entralpi Force Plate) was featured in two studies (24, 32) and is specifically designed for measuring various climbing-related parameters. A previous study (14) combined two measurement instruments: a climbing-specific dynamometer (Tindeq Progressor) and a generic force platform (Kistler Quattro).

“Key methodological aspects, such as testing position, grip type, and warm-up protocols, are summarized in Table 4”.

**Table 4:** Methodological aspects.

Study	Protocol	Bilateral	Instruments
Morenas et al. (22)	MIFS	Yes/Both hands	Dynamometer. Pine wood
Watts and Jensen (23)	MIFS/ED	Yes	Dynamometer
Mcclean et al. (24)	MIFS/CF	No/Manual Dominance	Force Plate Entralpi; Wooden edge
Bergua et al. (25)	ED	Yes/Both hands	Adjustable wood edge
Orth et al. (26)	MIFS/RFD	Yes	Specific dynamometer
Levernier and Laffaye (27)	MIFS/RFD	No	Specific dynamometer
Michailov et al. (28)	MIFS/ED	No	3D Force sensor; Wooden edge
Torr et al. (6)	MIFS	Yes	Wooden hangboard Lattice Training.
Baláš et al. (29)	ED (NIRS)	No	3D-SAC dynamometer. Wooden edge
Baláš et al. (30)	MIFS	No	Wooden hangboard AIX
Baláš et al. (31)	MIFS	Yes	non specific dynamometer
Labott et al. (14)	MIFS	Yes	Dynamometer(Tindeq) and force plate. Wooden hangboard
Macdougall et al. (32)	MIFS	No	Force plate. Entralpi and Pasco. Wooden edge.
López Rivera et al. (33)	ED	Yes/Both hands	Adjustable depth wooden edge
Söderqvist et al. (7)	MIFS	No	Force plate;Wooden hangboard Beastmaker. Wooden edges

M, minutes; d, days; h, hours; s, seconds; RFD, rate of force development; MIFS, maximal isometric finger strength of finger flexors; ED, endurance; CF, critical force.

### 3.3 Variables of interest and key results

#### 3.3.1 Reliability

In this review, ICC values were interpreted as follows: values below 0.5, low reliability; values between 0.5 and 0.75, moderate reliability, values between 0.75 and 0.9, good reliability, and values above 0.9, excellent reliability (34). Because most studies did not specify the ICC model or form used, we extracted the main ICC values exactly as reported by the authors. When multiple ICCs were available for the same protocol, we selected either the value identified as the primary outcome or, if none was indicated, the median ICC across test conditions. This approach ensures methodological consistency while acknowledging the lack of standardized ICC reporting across studies.

ICC values showed considerable variability across studies, as each study employed different grip types and positions based on the diverse characteristics of climbing grips (21). Despite this variability, most studies (6, 14, 22–26, 28, 30–33) (twelve in total) demonstrated good to excellent reliability in their measurements, with ICC values exceeding 0.75. This indicates that the measurements were generally consistent and reproducible. The remaining three studies (7, 27, 29) displayed greater variability, with some tests yielding lower ICC values (low to moderate reliability). In one study (7), the lowest recorded value (F3 Left = 0.605) was attributed by the authors to potential measurement errors, suggesting that testing conditions may have influenced the consistency of the results. Another study (29) specifically evaluated the reliability of Near-Infrared Spectroscopy (NIRS) by simulating climbing grips through intermittent contractions.

To provide a clearer synthesis, ICC values were compared across key subgroups:

Grip type: ICCs were generally highest for standardized Half Crimp (HC) grips, particularly on 20–23 mm edges, with values frequently above 0.90.22,14,24 Open Hand (OH) grips also produced high reliability when edge depth was standardized, although some variability was observed in studies allowing participants to select their grip (7).

Edge depth: Fixed edges of 20–23 mm consistently showed the most stable results, with ICC medians above 0.85 across multiple studies (6, 28). Variable edge depths produced more heterogeneous outcomes, especially when size was adjusted to individual morphology (23, 33).

Measurement devices: Electronic dynamometers and force platforms yielded higher ICC values compared to simpler setups using additional weights or non-instrumented edges (26, 30). This suggests that continuous force recording and standardized instrumentation improve measurement reliability.

Overall, ICC values across all studies ranged from 0.294 to 1.00, with a median of 0.86, highlighting good-to-excellent reliability when protocols and measurement conditions were standardized.

#### 3.3.2 Hand grip

There were no standardized hand grips used across studies; however, most selected one or more of the most representative climbing grips: Open Hand (OH), Half Crimp (HC), or Crimp (C) (35). In most studies (7, 14, 22–24, 26–32) (twelve), grip types were predetermined by the evaluators. In three studies (6, 25, 33), climbers were allowed to choose between OH or HC grips. At least the OH grip was included in eight studies (6, 22, 23, 25, 28, 30, 31, 33), and the HC grip was included in eight studies (6, 7, 22, 24, 25, 27, 32, 33) as well. Across these comparisons. ICCs were generally higher for standardized HC grips on edges of 20–23 mm.

#### 3.3.3 Edge depth

The reviewed studies showed significant variability in the grip sizes used for measurements, ranging from 6 to 60 mm. A majority (6, 7, 14, 24, 27–33) (eleven studies) employed fixed edge depths, whereas studies (22, 23, 26) used variable edge sizes adjusted based on the test requirements or participant characteristics. In one study (23), edge size was determined by the length of the climbers' proximal phalanges, while another study (22) did not specify the criteria for adjustment. One study (25) incorporated both fixed and variable edges for different measurements. Among the fixed sizes, 20 mm and 23 mm were the most frequently used, appearing in eight (6, 7, 14, 24, 28–30, 32) of the 15 studies. These depths consistently showed ICCs > 0.85, suggesting they may represent an emerging standard.

#### 3.3.4 Condition control

Most studies (6, 14, 22, 23, 26–29, 31, 32) did not control environmental conditions such as temperature, humidity, or chalk use on the day of the measurements. Four studies (24, 25, 30, 33) monitored temperature and humidity during test and retest sessions. One study (7) specifically referenced the use of chalk during testing to minimize finger moisture. Environmental conditions such as temperature, humidity, or chalk use were not consistently controlled across studies, with only four monitoring temperature and humidity and one explicitly reporting chalk use.

“Variables of interest and main outcomes, including reliability metrics and ICC values, are presented in Table 5”.

**Table 5:** Variables of interest and Key results.

Study	ICC	Rest	Edge depth	Hand grip	Conditions control
Morenas et al. (22)	0.99	24 h	(-);Variable	C, HC and OH(S)	NO
Watts and Jensen (23)	0.902- 0.947	60 s	(-); Variable	OH	NO
Mcclean et al. (24)	0.82–0.938	48 h	20 mm	HC	Yes
Bergua et al. (25)	0.89–1.00	7d	6–40 mm;Variable	OH or HC	Yes
Orth et al. (26)	0.83–0.93	7d	Variable	C	NO
Levernier and Laffaye (27)	0.95–0.98; 0.58–0.98	10–12d	10 mm	HC and S	NO
Michailov et al. (28)	0.92–0.98	7d	23 mm	OH	NO
Torr et al. (6)	0.91–0.98	48 h	20 mm	OH or HC	NO
Baláš et al. (29)	0.294–0.692	3–6d	23 mm	(C)^a^	NO
Baláš et al. (30)	0.88–0.94	6–7d	23 mm	OH, C, IM and MR	Yes
Baláš et al. (31)	0.95–0.98	(-)	10 mm	OH	NO
Labott et al. (14)	0.90–0.98	15 m	20 mm	C	NO
Macdougall et al. (32)	0.991–1	(-)	20 mm	HC	NO
López Rivera et al. (33)	0.88–0.97	7d	6–14 mm	OH or HC	Yes
Söderqvist et al. (7)	0.605–0.96	1–7d	20 mm	F3, HC, C, 35S, and two different pinch grips	NO/Use Chalk

^a^ The specific type of grip is not explicitly indicated; however, reference is made to the grip that maximizes activation of the flexor digitorum profundus muscle: C (36).

Mm, milmeters; HC, half crimp; S, slope; OH, open hand; C, full crimp and crimp; IM, index plus middle finger; MR, middle plus ring finger; F3, front 3 drag; 35S, 35-degree sloper.

## 4 Discussion

This systematic review evaluated the reliability of finger flexor strength assessment methods for climbers. The key findings were as follows:

1. Good to excellent reliability was observed in 14 of the 15 studies analyzed, with excellent reliability reported in all maximal isometric finger strength (MIFS) tests.
2. Limited specificity of the tests, as most were conducted either unilaterally or bilaterally in a non-simultaneous manner, which does not align with the motor patterns typical of climbing.
3. Consensus regarding the contact material for fingers in eleven of the fifteen studies, suggesting some standardization in this aspect of the methodology.

### 4.1 Instruments

A significant diversity of instruments for evaluating finger flexor strength among climbers was identified. This variability has important implications for the precision and comparability of measurements across studies.

Seven studies (22, 23, 26–29, 31) used dynamometers or force sensors specifically adapted for climbing assessment. These instruments are critical for providing precise and reproducible measurements and for generating detailed data on maximal isometric strength and the rate of force development. Two studies (14, 28) used climbing-specific dynamometers, one of which, the Tindeq Progressor, is widely used among advanced climbers. These sensors allow not only the quantification of maximal strength but also the assessment of dynamic parameters related to climbing biomechanics. Such technological advances are particularly valuable for studies aiming to understand the physical determinants of climbing performance in greater depth. Four studies (7, 24, 30, 32) employed force platforms or electronic scales, such as the Entralpi Force Plate, which was featured in two studies (24, 32). This instrument, designed for functional climbing strength evaluation, enables measurements of load distribution in multiple planes and under dynamic conditions, for example between both hands, and allows researchers to explore how external factors might influence performance (7, 24). Additionally, these force platforms can be used in combination with specialized devices, as seen in one study (14), which combined the Tindeq Progressor dynamometer with a generic force platform (Kistler Quattro). However, some studies did not take advantage of these technological advances. Three studies (6, 25, 33) did not use dynamometers or electronic devices; instead, they relied on less precise methods, such as fixed wooden grips or additional weights for climbers (6, 25, 33). Unlike dynamometers, which automatically account for body weight, record continuous force–time data, and capture variables like rate of force development, these simpler methods only register external load and lack the precision needed to detect small performance differences. Although inexpensive and accessible, their limited sensitivity reduces comparability with results obtained from advanced instruments. One study (22) stands out for its unique approach to measuring the load exerted by each finger individually. This was achieved by integrating a dynamometer into the grip edge and varying the hand position for each measurement, providing individual finger load values within the overall grip.

Wooden edges or contact surfaces were reported in 11 studies (6, 7, 14, 22, 24, 25, 28–30, 32, 33), wood is widely used in climbing research because it offers realistic tactile properties, simulates common grip types, and remains cost-effective (30). However, four studies (23, 26, 27, 31) did not report the contact material used, which may have introduced variability. Research shows that surface material can slightly influence friction and load distribution between (37). Future studies should consistently report this variable and consider standardized materials to improve comparability rather than assuming negligible effects.

Finally, there is still a lack of testing methods that truly reflect the demands of climbing and address the challenge of ecological validity, as laboratory settings rarely replicate the complexity and variability of real climbing (38).

### 4.2 Bilateral measurements

The lack of specificity in most tests stands out, with non-simultaneous measurements (using only one arm) being conducted in 12 studies (6, 7, 14, 23, 24, 26–32) While sport climbing rarely involves hanging by a single arm, whether bilateral testing better reflects real climbing demands remains uncertain and requires further investigation rather than immediate application (12, 39). Single-arm assessments could still be valuable if performed in safer, non-hanging positions—such as the mid-thigh pull—that allow maximal force measurement without excessive joint loading or technical demands. Notably, three studies (22, 25, 33) conducted measurements using both arms simultaneously, providing an opportunity for future research to clarify whether bilateral protocols improve ecological validity or injury risk assessment.

### 4.3 Reliability

Most studies (6, 7, 14, 22–28, 30–33) reported ICC values above 0.75, with significantly higher values observed in tests assessing maximal strength. These results confirm the generally good-to-excellent reliability of the protocols analyzed. Conversely, two studies (7, 27) reported lower ICC values (0.58 to 0.605) in certain tests, mainly due to measurement errors and the inclusion of non-climbers, which introduced variability not present in athlete-only samples. In one study (27), ICC variations were observed, with the lowest values occurring in measurements of Rate of Force Development (RFD). The non-climber group exhibited the largest discrepancies, suggesting that the test conditions and participant characteristics can affect measurement accuracy. Another study (29) reported significantly lower ICC values (0.294 to 0.692), attributed to limited variability among participants, as reduced between-subject variance in homogeneous samples can artificially lower reliability coefficients even when measurement error remains constant. A further factor contributing to reduced reliability in this study was the alteration of the Tissue Saturation Index (TSI) during warm-up.

Although Studies with higher CAT scores tended to report better reliability, this relationship was not consistent across all protocols, suggesting that methodological rigor alone does not fully explain ICC differences. Overall, the heterogeneity in devices, edge depths, and grip protocols requires cautious interpretation rather than direct comparison.

A narrative synthesis showed that the most reliable results came from standardized Half Crimp grips on fixed edges of 20–23 mm (22, 24), while variable edge depths or self-selected grips led to greater variability (7). Similarly, studies using electronic dynamometers or force platforms reported more consistent ICC values than those relying on wooden grips or external weights, underscoring the importance of standardized instrumentation and controlled testing conditions. Across all studies, ICC values ranged from 0.294 to 1.00, with a median of 0.86, confirming that reliability was generally good to excellent when protocols were carefully standardized.

### 4.4 Hand grip and edge depth

One of the primary sources of heterogeneity among the studies was the variation in the handgrip and edge depths used. Although most studies (7, 14, 22–24, 26–32) (twelve) followed uniform protocols using climbing-representative grips, such as open hand (OH), half crimp (HC), or crimp (C), no standard exists for grip selection. Eight studies (6, 22, 23, 25, 28, 30, 31, 33) included the OH grip in their tests, while another eight (6, 7, 22, 24, 25, 27, 32, 33) incorporated the HC grip. The two hanged grip types are commonly used in climbing development. In three studies (6, 25, 33), participants were allowed to select their hand grip based on their preference or experience (OH or HC), introducing an additional source of bias in the measurements. One study (27) noted that the Crimp (C) grip generated greater force than the half crimp (HC) grip, although it also increased the risk of injury.

There were substantial differences in the edge depths used across studies. Notably, eight studies (6, 7, 14, 24, 28–30, 32) employed fixed grip depths of 20 and 23 mm. In one study (30), these sizes were considered as ideal for minimizing pain during gripping. Another study (33) used various depths (6, 8, 10, 12, and 14 mm) to determine the optimal grip size for evaluating the maximum suspension time. Studies using variable grip sizes (22, 23, 26) adopted different criteria based on test requirements or participant finger sizes. Despite this variability, a general trend was observed: grips of 20 and 23 mm were predominantly used for MIFS, while smaller or variable grips were used for assessing other capacities. An exception was a study (31) which used a 10 mm grip size for MIFS measurement. This suggests that smaller grip depths may be better suited for evaluating endurance, while larger depths (20 mm or more) are preferable for measuring the maximal strength and rate of force development (RFD).

### 4.5 Condition control

Only four (24, 25, 30, 33) of the 15 evaluated studies explicitly controlled environmental conditions, such as temperature and humidity, to ensure stable settings between test and retest sessions. Another study (7) recommended using chalk during tests, which improves the friction coefficient according to some research (40). The lack of systematic control in most studies may have introduced variability related to external factors, such as humidity effects on grip strength or differences in muscle fatigue associated with temperature. Minor fluctuations in muscle or ambient temperature can influence force generation and contractile efficiency (41, 42). Specifically, it has been demonstrated that cold ambient temperatures detrimentally affect climbing-specific finger flexor performance, particularly reducing muscular endurance (43). Additionally, although chalk is widely used to reduce finger moisture, under certain conditions it may actually decrease the friction coefficient (44). The absence of standardized control over these environmental and frictional variables thus represents a significant source of heterogeneity across studies.

### 4.6 Demographic distribution and representativeness

A total of 747 participants from 15 studies were included, representing a sample of climbers with diverse characteristics and skill levels, ranging from IRCRA levels 3 to 29. This heterogeneity limits the generalizability of the findings and underscores the need for more balanced designs, especially regarding sex-specific and level-specific analyses.

This wide range of abilities offers the possibility of exploring a broad spectrum of capabilities, from beginner to elite climbers. However, this variability may influence results since finger flexor strength differs significantly between novice and elite climbers (7). Additionally, one study (27) included a control group of nine non-climbers and did not provide information regarding the gender of the participants. This introduces a potential source of variability, as the physical and biomechanical characteristics of non-climbers can differ significantly from those of climbers, complicating the analysis of potential differences in strength measurements between males and females. Another study (32) did not specify the skill levels of its participants, further limiting its ability to interpret findings in relation to specific climbing demands.

These gaps in participant characterization are significant because gender and athletic experience affect testing outcomes (15, 45). Moreover, sample sizes across the studies ranged from 13 to 244 participants, highlighting a significant imbalance in statistical power. Studies with smaller sample sizes, such as those with 13 participants (24), are more susceptible to stochastic errors and selection bias, which may render the conclusions less reliable. In contrast, the study with the largest sample size (6) (244 participants) could provide more robust and generalizable results, although intragroup heterogeneity might obscure specific differences between subgroups.

### 4.7 Study limitations

The main limitation of this review is the considerable heterogeneity among the included studies, particularly regarding methodologies, measurement instruments, grip types, edge depths, and testing conditions. This variability complicates direct comparisons and makes meta-analysis inappropriate, as pooling heterogeneous protocols would undermine the validity of the results.

In addition, limiting the search to English and Spanish publications and excluding gray literature may have introduced selection bias, as studies with non-significant results are often underrepresented in peer-reviewed journals. Consequently, the possibility of publication bias cannot be fully dismissed.

Differences in participant skill levels may also have influenced reliability outcomes, with elite climbers frequently showing greater measurement consistency, likely due to their experience with testing protocols. Finally, the lack of standardized procedures for edge size, grip type, and environmental control across studies reduces comparability and hinders the development of unified testing guidelines.

### 4.8 Future directions

This review underlines the need to move toward common standards in climbing strength assessments. One practical step is the design of devices that are easy to reproduce, affordable, and specific to climbing so they can be applied both in research and in everyday training. A promising approach could be the use of 3D-printed PLA mixed with wood fibers; this material is light, durable, and has a tactile quality close to natural wood, while also being simple to replicate. There is still a clear need to agree on common testing common reference protocols for finger strength in climbing. Several studies point out that edges of around 20–23 mm provide a demanding but safe stimulus and allow results to be compared across research (46, 47). Testing each hand separately also seems more useful, as bilateral protocols may mask asymmetries; dedicated unilateral testing could better quantify interlimb differences (48).

Grip position is another issue. The open-hand posture, although generally considered safer, does not always fit the natural shape of the fingers. On flat edges, it often produces compensatory bending at the proximal interphalangeal joints, which reduces the comparability of data (49). For this reason, exploring stepped or anatomically shaped edges should be considered a hypothesis for future validation rather than a conclusion from the present synthesis. These designs could, in theory, improve safety, ecological validity, and measurement reliability, but further empirical evidence is needed before making definitive recommendations.

## 5 Practical applications

The findings of this review are directly relevant for climbers and their coaches. Among the protocols analyzed, maximal isometric finger strength (MIFS) assessments using fixed edges of 20–23 mm with standardized grip positions showed the highest reliability, making them the preferred option for both research and training applications. Tests of maximal isometric finger strength (MIFS) have repeatedly shown high reliability, which makes them a solid option for tracking strength gains at different performance levels.

In practice this means that test conditions need to be kept stable. Small details -temperature in the room, humidity, or how chalk is used- can change the results. Using the same grip type each time, and being consistent when testing one hand or both, also helps. For elite climbers, single-arm tests in controlled positions (e.g., mid-thigh pull) may help detect asymmetries and fine-tune training, whereas recreational climbers may benefit from simpler bilateral protocols to monitor general strength progression while minimizing technical demands. With these precautions, assessments become more trustworthy, training can be adjusted more precisely, and the risk of injury is reduced. In the end, the goal is to make sure that test results reflect as closely as possible the demands of real climbing.

## 6 Conclusion

This systematic review emphasized the reliability of methods for evaluating finger flexor strength in climbers, demonstrating good to excellent reliability across most reviewed studies, with excellent reliability in all MIFS measurements. However, methodological heterogeneity and the lack of standardization in various aspects pose significant challenges to the generalization and applicability of the findings. These issues limit the comparability of results and the extrapolation of conclusions. The variability in handgrip, edge depths, and the lack of environmental control emphasize the urgent need for standardized guidelines to ensure methodological consistency in future research.

Differences in participant characteristics further underscore the necessity of designing future studies with greater homogeneity in sample selection and a further detailed description of individual demographic and athletic traits. Among the 15 studies examined, the high reliability revealed in this review, classified as good to excellent (ICC > 0.75), supports confidence in the measurements of maximal isometric finger strength. Thus, these tests can be considered reliable tools for climbing research. Nonetheless, variability observed in one study, in which reliability values (ICC = 0.58–0.605) were significantly lower, has been attributed to factors such as the inclusion of non-climber participants and the inadequate control of experimental conditions. Moreover, reduced between-subject variability in homogeneous samples likely contributed to artificially low ICC coefficients, given the sensitivity of this metric to total variance. These results indicate that although reliability is generally high, heterogeneity in participant profiles and testing protocols can affect measurement precision. Standardization of methodologies and control of test conditions are essential for future research.

Additionally, important aspects such as the separation or proximity of the fingers during measurements and the angle of the edge used (e.g., 90 degrees or less) remain unspecified in the reviewed studies, introducing a lack of standardization in these critical parameters. Future work should also focus on validating these protocols in female and youth climbers, as well as on developing testing procedures that better replicate the physical demands of real climbing scenarios to improve ecological validity.

These findings underscore the urgent need to standardize strength measurement protocols for climbers. This includes a clear definition of grip types and edge depths, as well as the establishment of controlled testing conditions. Without these consensus-based guidelines, meaningful comparisons across studies and the development of evidence-based training and injury-prevention strategies will remain limited.

## Data Availability

The datasets generated and analyzed during the current study are available in the article and its Supplementary Material.
